# Baicalin Attenuates Oxidative Stress in a Tissue-Engineered Liver Model of NAFLD by Scavenging Reactive Oxygen Species

**DOI:** 10.3390/nu14030541

**Published:** 2022-01-26

**Authors:** Wen Gao, Bin Xu, Yizhi Zhang, Shuang Liu, Zhongping Duan, Yu Chen, Xiaohui Zhang

**Affiliations:** 1 Department II of Liver Diseases, Beijing Youan Hospital Affiliated to Capital Medical University, Beijing 100069, China; gaowen34331837@126.com (W.G.); xubin1016@126.com (B.X.); 2 Beijing Key Laboratory of Liver Failure and Artificial Liver Treatment, Department IV of Liver Diseases, Beijing Youan Hospital Affiliated to Capital Medical University, Beijing 100069, China; zhangyizhi2020@163.com (Y.Z.); shuangliu186@163.com (S.L.); duan@ccmu.edu.cn (Z.D.); 3 Beijing Advanced Innovation Center for Big Data-Based Precision Medicine, Capital Medical University, Beijing 100069, China

**Keywords:** baicalin, nonalcoholic fatty liver disease, tissue-engineered liver, oxidative stress

## Abstract

Oxidative stress plays an important role in the pathogenesis of nonalcoholic fatty liver disease (NAFLD). Baicalin has been shown to exert protective effects in various liver diseases. The mechanism of baicalin’s antioxidative effect in NAFLD is currently unclear. The aim of this study was to investigate the effects and mechanisms of baicalin on oxidative stress in a new tissue-engineered liver model of NAFLD. The 3D model of NAFLD was induced by a fat-supplemented medium (fatty acids, FFA group) for 8 days and baicalin was administered on the 5th day. CCK-8 assay showed that baicalin at concentrations below 100 μM had no obvious cytotoxicity. Baicalin inhibited apoptosis and lactate dehydrogenase release in the FFA group. Baicalin reduced the levels of reactive oxygen species and malondialdehyde induced by FFA, and increased superoxide dismutase and glutathione amounts. However, it did not upregulate nuclear erythroid 2-related factor 2 compared with the FFA group. Mitochondrial morphology was partially restored after baicalin treatment, and ATP5A expression and mitochondrial membrane potential were increased. The superoxide anion scavenging ability of baicalin was enhanced in a dose-dependent manner. In summary, baicalin reduces oxidative stress and protects the mitochondria to inhibit apoptosis in the 3D NAFLD model via its own antioxidant activity.

## 1. Introduction

Nonalcoholic fatty liver disease (NAFLD) is characterized by lipid accumulation in hepatocytes and considered to be the liver manifestation of metabolic syndrome [1]. The pathological range of NAFLD varies from simple nonalcoholic fatty liver to nonalcoholic fatty hepatitis (NASH), cirrhosis and even to hepatocellular carcinoma [2,3]. Oxidative stress plays a vital role in the pathogenesis of NAFLD [4]. It results from an imbalance between pro-oxidants and antioxidants that cause damage to cells and other macromolecules, including proteins [5,6]. The main pro-oxidant chemicals in fatty liver include superoxide anion, hydrogen peroxide and hydroxyl radical, which are collectively referred to as reactive oxygen species (ROS). Fatty acid oxidation is an important source of reactive oxygen species in fatty liver [7]. The excess fat and ROS in the liver cause lipotoxicity and lead to mitochondrial dysfunction, inducing damage in hepatocytes and inflammation [8,9,10]. Therefore, NAFLD is the most common cause of cryptogenic cirrhosis.

Currently, developing a safe and effective pharmacological treatment option for NAFLD/NASH remains challenging. Baicalin (C_21_H_18_O_11_) is an active flavonoid originating from the root of *Scutellaria baicalensis* Georgi, which exerts anti-inflammatory and antioxidant effects in liver diseases [11]. Baicalin has various pharmacological activities, including antitumor, antimicrobial and antioxidant effects, with wide clinical applications [12,13,14]. However, whether baicalin has protective effects on hepatocyte lipotoxicity and the associated mechanism remain elusive. In this study, a 3D model of tissue-engineered fatty liver was used to investigate the effects and mechanisms of baicalin in NAFLD, mainly focusing on antioxidation and mitochondrial function.

## 2. Materials and Methods

### 2.1. Cells and Reagents

HepG2 cells were purchased from the American Type Culture Collection (ATCC, Manassas, VA, USA). Fetal bovine serum, DMEM, pancreatin and penicillin were purchased from Gibco (Suzhou, China). Peristaltic pumps were available from MasterFlex (USA). PES membrane filters (0.22 μm, Millex-GV) were purchased from Merck Millipore Ltd.); intravenous indwelling needles (BD Intima II) were from Becton Dickinson medical Devices Co. Ltd. (Suzhou, China). Oleic acid, palmitic acid and sodium deoxycholate were all obtained from Sigma (San Francisco, CA, USA). The protein quantitation kit and the mitochondrial membrane potential detection kit (JC-1) were obtained from Beyotime Biotechnology Co. Ltd. (Shanghai, China). Sodium deoxycholate (SDC) and phospholipase A2 were obtained from Sigma company (Beijing, China). Detection kits for triglyceride (TG), malondialdehyde (MDA), reactive oxygen species (ROS), glutathione (GSH) and superoxide dismutase (SOD) were obtained from Nanjing Jiancheng Bioengineering Research Institute (Nanjing, China). The total antioxidant capacity assay kit with the rapid ABTS method and the superoxide anion scavenging capacity test kit were obtained from Solarbio science & technology co. Ltd. (Beijing, China). Baicalin, the main component of Shuganning Injection, was provided by Guizhou Ruihe Pharmaceutical Co. Ltd.

### 2.2. Fat-Supplemented Medium Preparation

The fat-supplemented medium was composed of DMEM with free fatty acids (FFAs) [15]. Briefly, OA and PA (2:1) were mixed with 10 M NaOH and transferred to an ultrasonic water bath at 60 °C for 30 min. Secondly, bovine serum albumin without fatty acids was added, and the final pH was adjusted to 7.4 with HCl. The filtered solution (0.22 μm filter) was added to the complete medium to make the fat-supplemented medium.

### 2.3. Establishment of a 3D NAFLD Model (Tissue-Engineered Fatty Liver)

**Figure 1:**
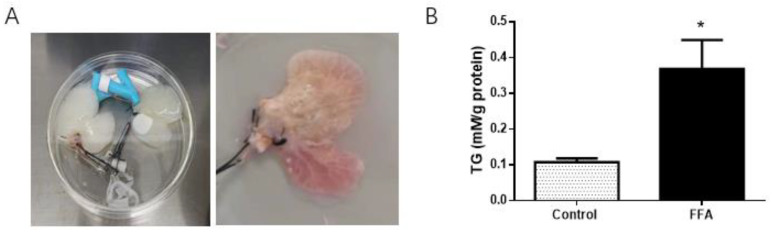
Establishment of a 3D model of NAFLD based on the tissue-engineered liver. (**A**) The process of model preparation. (**B**) Intracellular TG measurement of the FFA group liver. * *p* < 0.05.

The NAFLD model of tissue-engineered fatty liver was prepared as previously described [16]. Briefly, the livers of male Sprague Dawley rats (200–230 g) were decellularized with 1% SDC buffer (including 20 μL/L phospholipase A_2_) and isolated after anesthesia as a natural liver scaffold. A total of 30 million HepG2 cells were seeded in scaffold for 3 times, and perfused with complete medium with a bioreactor. Bioreactor parts were assembled under a sterile hood according to Figure 1 and placed in an incubator with 5% CO_2_ at 37 °C. After 24 h of seeding, the fat-supplemented medium was used instead of complete medium to induce the tissue-engineered fatty liver. The culture medium was changed every day, and baicalin was administered at 5 days after replacing the fat-supplemented medium for 3 days. The tissue-engineered liver, which was cultured with complete medium for 8 days, was used as a control.

### 2.4. Hematoxylin and Eosin (H&E) Staining and Immunofluorescence

Liver samples were fixed with 4% formaldehyde overnight at 4 °C and submitted to gradient dehydration. After paraffin embedding, the sections were sectioned at 7 μm for H&E staining. For immunofluorescent staining, liver tissue samples were embedded in Optimum Cutting Temperature Medium (Sakura Finetek USA Inc., Torrance, CA, USA), and sectioned at 7 μm. The sections were fixed with 4% formaldehyde for 20 min and washed three times with PBS. Then, they were incubated with 0.12% Triton X-100 for 10 min, and blocked with 10% goat serum for 1 h at room temperature. Then, the samples were incubated with ATP5A monoclonal antibody (Invitrogen, Rockford, IL, USA) overnight at 4 °C. After washing with PBS, the sections were incubated with goat polyclonal secondary antibodies against mouse IgG-H&L (Alexa Fluor^®^ 647, Abcam, Cambridge, UK) for 1 h at room temperature. Following the final wash, the sections were covered with coverslips and examined under a fluorescence microscope (Nikon, Tokyo, Japan). The negative control was incubated with secondary antibodies alone.

### 2.5. Cell Viability/Cytotoxicity Assay

HepG2 cells were cultured in 96-well plates with complete medium or fat-supplemented medium, and treated with different concentrations of baicalin for 24 h or 48 h. After treatment, CCK-8 working solution (Dojindo, Japan) was added into each well, and the plate was incubated for 30 min at 37 °C. Absorbance (OD) at 450 nm was measured on a microplate spectrophotometer (Bio-Rad, Hercules, CA, USA), and the rate of cells alive was calculated relative to controls (%).

### 2.6. Lactate Dehydrogenase (LDH) Measurement

LDH was used to evaluate liver function and the degree of cell damage. The culture supernatants of the tissue-engineered fatty liver samples incubated with or without baicalin were collected every day and sent to the Clinical Laboratory at Capital Medical University (Beijing, China) for LDH detection.

### 2.7. Intracellular TG Measurements

Cells in the 3D-cultured system were harvested by digestion with type IV collagenase (0.25 mg per 100 mL), washed with PBS and lysed for intracellular TG measurement according to the manufacturer’s instructions (Nanjing Jiancheng Bioengineering Institute, Nanjing, China). Absorbance in each cell homogenate was measured at 450 nm on a microplate spectrophotometer (Bio-Rad). The final TG content was calculated and presented as mM/g protein.

### 2.8. Western Blotting

Cells isolated from 3D-cultured livers were washed twice with PBS, followed by protein extraction with RIPA solution containing a protease inhibitor cocktail (1:50, Roche). Protein concentrations were assessed with the bicinchoninic acid (BCA) Protein Quantification kit. Proteins (60 μg) were separated by SDS-PAGE and transferred onto polyvinylidene fluoride membranes. After blocking with 10% milk for 1 h, the membranes were incubated with rabbit primary antibodies against cleaved caspase-3 (Cell Signaling Technology, Danvers, MA, USA) and erythroid 2-related factor 2 (Nrf2) (Cell Signaling Technology), followed by incubation with species-matched secondary antibodies (Anti-rabbit IgG, HRP-linked Antibody; Cell Signaling Technology). Immunoreactive bands were detected by enhanced chemiluminescence (Thermo Fisher Scientific, Rockford, IL, USA) and developed by exposure on a transilluminator (Bio-Rad). β-actin was used as an endogenous control.

### 2.9. Apoptosis Detection

Harvested cells or frozen sections were fixed with 4% paraformaldehyde for 15 min, and washed with PBS after incubation with 2% Triton for permeabilization. Then, the cells or sections were incubated at 37 °C for 60 min with 50 μL TUNEL staining solution (Roche, Mannheim, Germany) in the dark and detected on a fluorescence microplate reader or observed by fluorescence microscopy.

### 2.10. Mitochondrial Membrane Potential Detection

Freshly isolated cells and JC-1 working solution (5 μg/mL) were mixed and incubated at 37 °C in the dark for 30 min. Then, the mixture was centrifuged for 4 min, and washed with JC-1 staining buffer. Fluorescence intensity was determined on a fluorescence microplate reader (Bio Tek Instrument Inc., VT, US) at Ex/Em = 485/525 nm (monomers) and Ex/Em = 525/590 (aggregates), respectively. MMP was calculated as the ratio of red (aggregates) to green (monomers) signals.

### 2.11. Transmission Electron Microscopy (TEM)

Fresh specimens were fixed with 5% glutaraldehyde and sent to the electron microscopy laboratory of Peking University health science center. After processing, the tissues were examined using a JEOL 1011 transmission electron microscope (JEOL, Tokyo, Japan).

### 2.12. Detection of Chemicals Associated with Oxidative Stress

For ROS measurement, fresh cells collected from the tissue-engineered liver were resuspended with DMEM containing 10 μM DCFH-DA (Nanjing Jiancheng Bioengineering Research Institute., Nanjing, China) at 37 °C for 45 min. After washing and resuspension with PBS, cells were assessed on a fluorescence microplate reader (Bio Tek Instrument Inc., Winooski VT, USA). Fresh or frozen cells from the tissue-engineered liver were also used for the measurements of MDA, SOD and GSH levels. All assays were performed in accordance with the instructions of respective kits, and absorbance was read on a microplate reader. The final results were normalized to protein concentration.

### 2.13. Detection of Antioxidant Capacity

The total antioxidant capacity (rapid ABTS method) and superoxide anion scavenging ability of baicalin were measured with respective commercial kits (Solarbio science & technology co., Ltd., Beijing, China). Different concentrations of baicalin (0.1 μM to 1 mM) were assessed, strictly according to the respective instructions. A microplate reader was used for absorbance reading at 405 nm or 530; the final data were presented as percentage (%).

### 2.14. Statistical Analysis

Statistical analysis and graphing were performed with GraphPad Prism 6.0 (GraphPad Software Inc., San Diego, CA, USA). Data are mean ± SEM, and were compared by one-way ANOVA for multiple comparisons or Student’s *t*-test for group pair comparisons. *p* < 0.05 was considered statistically significant.

## 3. Results

### 3.1. Successful Establishment of a 3D NAFLD Model

A 3D NAFLD model in vitro was established using human HepG2 cells subcultured on rat liver biological matrix scaffolds, which were perfused with fat-supplemented medium (FFA group). The tissue-engineered liver cultured with a normal medium was used as a control (Figure 1A). Intracellular TG content in the FFA group was 2.4-fold that of the control group (*p* < 0.05, Figure 1B) at 8 days, indicating the NAFLD model was successfully established.

### 3.2. Baicalin Reduces Cell Damage in the FFA Group

**Figure 2:**
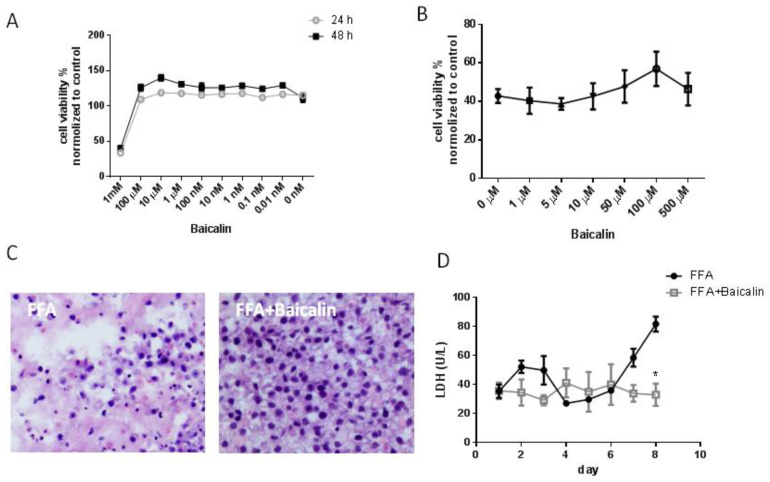
The protective effect of baicalin on hepatocytes cultured with fat-supplemented medium. (**A**) Cytotoxicity of baicalin in HepG2 cells treated for 24 h and 48 h (CCK8 assay, *n* = 6). (**B**) After cells were cultured with fat-supplemented medium, the effects of different concentrations of baicalin on cell viability were detected (CCK8 assay, *n* = 6). (**C**) H&E staining showed pathological changes in the FFA group with or without baicalin treatment (200×). (**D**) LDH levels in the supernatant of each group were measured every day (*n* = 5). * *p* < 0.05.

First, we determined the safe concentration of baicalin. The CCK-8 assay showed that baicalin at 0.01 nM to 100 μM had no obvious cytotoxicity in HepG2 cells both at 24 h and 48 h; at 1 mM, cell viability was less than 50% (Figure 2A). A high concentration of fat-supplemented medium induced hepatocyte injury. In HepG2 cells cultured with fat-supplemented medium (monolayer culture), baicalin treatment gradually increased cell viability with concentration changed from 10 μM to 100 μM (Figure 2B). Therefore, we chose 100 μM to investigate the effects of baicalin in subsequent experiments.

In the FFA group (3D culture), baicalin (100 μM) also showed a protective effect on hepatocytes. H&E staining showed there were large amounts of damaged cells in the FFA group, presenting cellular shrinkage, nuclear pyknosis and nuclear fragmentation; baicalin treatment largely reduced cell death (Figure 2C). The protective effect of baicalin was also evidenced by LDH measurements. After incubation with baicalin (100 μM), LDH release in the FFA group was decreased by 59.7% on day 8 compared with no treatment (*p* < 0.05, Figure 2D), indicating that baicalin inhibited cell injury induced by FFA and restored liver function to a certain extent.

### 3.3. Baicalin Attenuates Oxidative Stress Process

**Figure 3:**
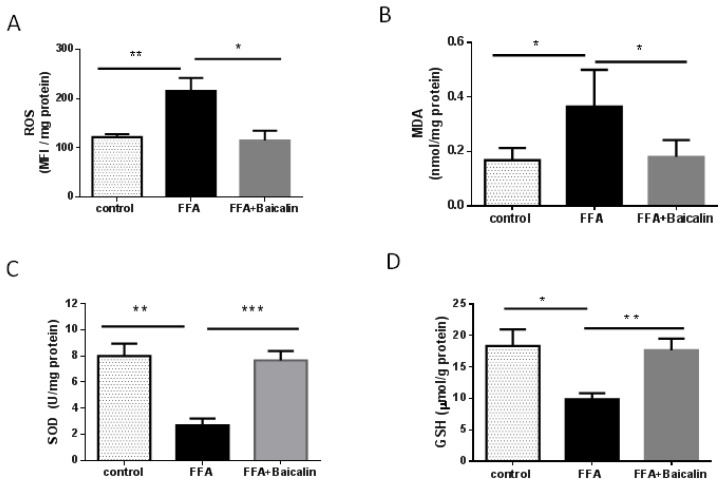
Regulatory effect of baicalin on the balance between oxidation and antioxidation. (**A**) ROS contents in each group. Data were presented as mean fluorescence intensity (MFI) per mg protein (*n* = 5). (**B**) MDA levels in each group (*n* = 6). (**C**) SOD amounts in each group (*n* = 5). (**D**) GSH levels in each group (*n* = 5). * *p* < 0.05; ** *p* < 0.01; *** *p* < 0.001.

In the animal model of NASH, the increase in ROS production in the presence of excess FFA has been verified. Oxidative stress, especially lipid peroxidation, induces liver injury and hepatic stellate cell activation. [17]. Therefore, we tested select indicators of oxidative stress. The levels of ROS in the FFA group were 2-fold those of controls (*p* < 0.01), and baicalin treatment significantly reduced ROS levels close to control levels (*p* < 0.05, Figure 3A). MDA is an end product of lipid oxidation, which could cause cross-linking polymerization of proteins and nucleic acids, showing cytotoxicity [18]. MDA alterations in different groups paralleled ROS changes, suggesting baicalin reduced ROS and oxidative stress-associated lipid toxic products (Figure 3B). On the contrary, baicalin increased intracellular SOD and GSH levels, which are representative antioxidants, compared with the FFA group (*p* < 0.01, Figure 3C,D).

### 3.4. Protective Effect of Baicalin on the Mitochondria

**Figure 4:**
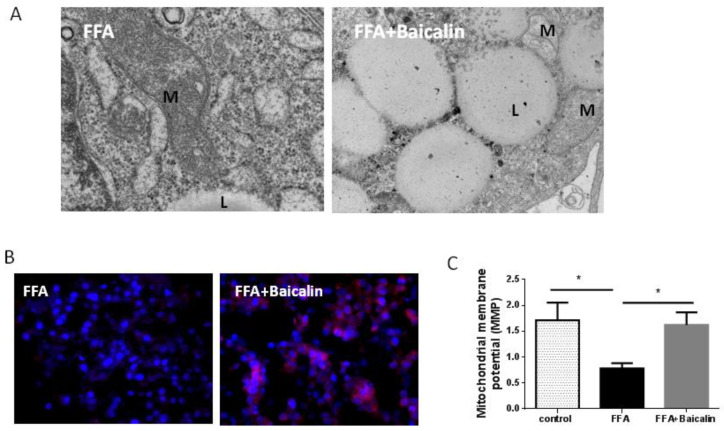
Ultrastructural morphology and function of mitochondria with or without baicalin treatment. (**A**) Mitochondrial morphology was observed by transmission electron microscopy. Lipid droplet (L), mitochondrial (M). (**B**) Expression of ATP5A-positive cells (red) in the FFA and baicalin groups. DAPI was used for counterstaining. (**C**) MMP detected with the JC-1 fluorescence probe (*n* = 5). * *p* < 0.05.

Mitochondrial dysfunction involves structural and metabolic damage, especially in oxidative stress-related diseases [19]. ROS produced during lipid metabolism are harmful to the mitochondria [20]. In the ultrastructure of samples from the FFA group, there were few visible mitochondria in the cytoplasm; in addition, mitochondrial morphology and membrane integrity were damaged, with multiple lipid droplets. Baicalin treatment increased the amounts of observed mitochondria and partly restored mitochondrial morphology, and sparse mitochondrial crista could be found in some mitochondria (Figure 4A). Further, we detected mitochondrial function. ATP5A is a key enzyme in oxidative phosphorylation (OXPHOS) and is responsible for energy production [21]. In the FFA group, the fluorescence intensity of ATP5A-positive cells was weak; however, baicalin obviously increased the expression of ATP5A (Figure 4B). Furthermore, MMP in the FFA group was reduced by 56.1% compared with controls (*p* < 0.05), and baicalin significantly reversed MMP close to control values (*p* < 0.05, Figure 4C. These results suggested baicalin partially restored mitochondrial function in the FFA group.

### 3.5. Baicalin Attenuates Apoptosis Induced by FFA

**Figure 5:**
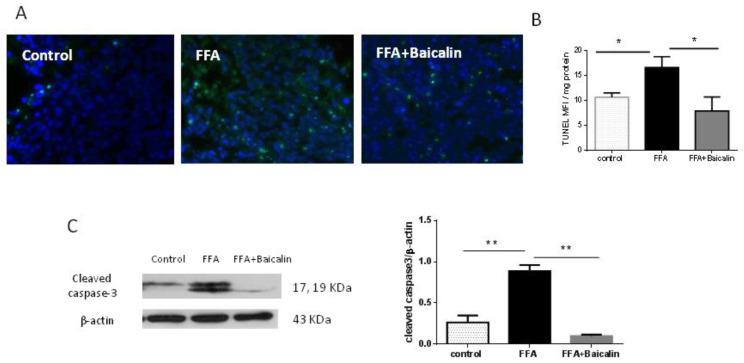
Baicalin decreases FFA-induced apoptosis. (**A**) TUNEL staining. TUNEL, green; DAPI, blue. (**B**) Quantitative analysis of TUNEL data; results were presented as MFI (*n* = 5). (**C**) Expression of cleaved caspase-3 among the three groups, assessed by Western blot. Immunoreactive bands are shown on the left, and quantitation is shown on the right (*n* = 5). * *p* < 0.05; ** *p* < 0.01.

Mitochondrial dysfunction activates the mitochondrial apoptotic pathway and leads to cell death [22]. In this study, there were great amounts of TUNEL-positive (green fluorescence) cells in the FFA group, and baicalin could reduce the amounts of TUNEL-positive cells (Figure 5A). Quantitative analysis of TUNEL showed that baicalin treatment significantly reduced fluorescence intensity compared with the FFA group (*p* < 0.05, Figure 5B). Furthermore, baicalin treatment significantly reduced cleaved caspase-3 protein amounts compared with the FFA group (*p* < 0.01, Figure 5C), suggesting that baicalin exerted a protective effect on FFA-induced hepatocyte apoptosis.

### 3.6. Antioxidant Activity of Baicalin

**Figure 6:**
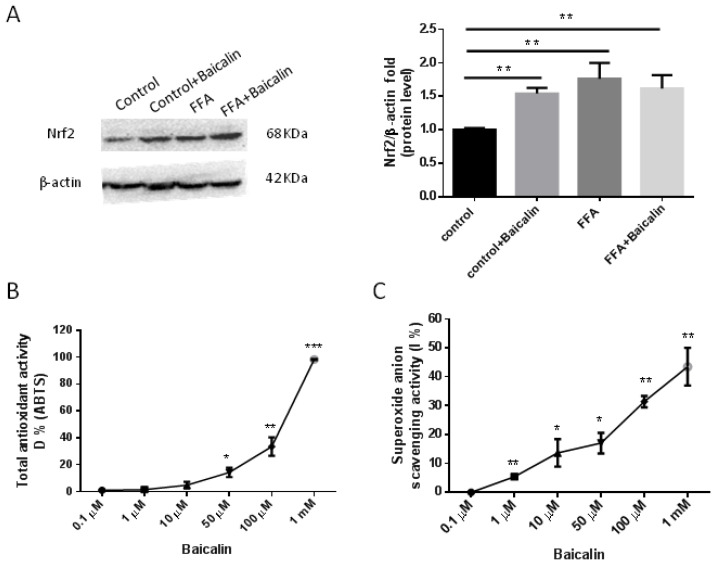
Antioxidant capacity of baicalin. (**A**) Representative Western blots for Nrf2 protein expression and quantification in each group (*n* = 6). (**B**) Total antioxidant capacity assessment by the ABTS method. (**C**) Superoxide anion scavenging activity assay. The concentrations of baicalin were from 0.1 μM to 1 mM. Duplicate experiments were repeated three times. All the values were compared with controls (0 μM baicalin), * *p* < 0.05; ** *p* < 0.01; *** *p* < 0.001.

Nuclear erythroid 2-related factor 2 (Nrf2) is a key regulator to prevent NASH, and mediates the antioxidant response by regulating glutathione metabolism [23]. Because of the antioxidant activity shown by baicalin in the above 3D NAFLD model, we detected Nrf2 expression in different groups. Western blot showed that in normal culture, baicalin significantly enhanced Nrf2 expression (*p* < 0.01; Figure 6A). In the FFA group, Nrf2 had higher expression compared with controls (*p* < 0.01; Figure 6A); after baicalin treatment, Nrf2 was still highly expressed, with no obvious difference compared to the FFA group (Figure 6A).

To examine the mechanism underlying the antioxidant effect of baicalin, we further detected the capability of baicalin itself to clear or neutralize ROS. In the total antioxidant capacity assay, baicalin at amounts higher than 10 μM had a certain antioxidant capacity in a dose-dependent manner (Figure 6B). In addition, we tested the scavenging activity of baicalin of superoxide anion (·O_2_^−^), a common ROS responsible for the promotion of oxidative stress under various pathophysiological conditions. Baicalin showed obvious scavenging activity in a dose-dependent manner (Figure 6C). The above findings indicated baicalin had certain antioxidant effects due to its molecular chemical structure.

## 4. Discussion

**Figure 7:**
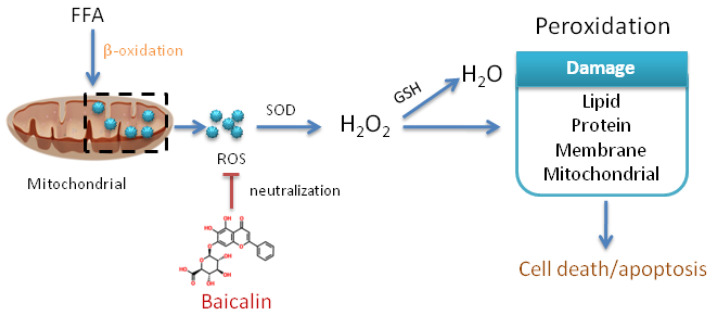
Summary of baicalin attenuating FFA-induced oxidative stress by scavenging reactive oxygen species. β-oxidation is the major oxidative pathway for fatty acids and a major source of ROS in mitochondria. ROS is converted to H_2_O_2_ under the action of SOD, and then H_2_O_2_ is decomposed into non-toxic H_2_O under the action of GSH. A lot of FFA leads to the imbalance between oxidation and antioxidation, producing excessive H_2_O_2_, which can cause damage to fat, protein, cell membrane and mitochondria, finally leading to cell death or apoptosis. Baicalin directly neutralizes ROS and reduces cell damage from oxidative stress, finally decreasing cell death/apoptosis.

Baicalin is found as a glycoside in *Scutellaria baicalensis* Georgi, whose roots have been extensively used as a traditional medicine in many East Asian countries to reduce inflammation. Previous studies have shown baicalin possesses a wide range of biological and pharmacological functions, including antiinflammation, anticancer and antipruritic effects [24]. This study firstly found baicalin attenuated FFA-induced apoptosis to exert cell protection in the NAFLD model through its own antioxidation (Figure 7).

In the current study, a new 3D model of NAFLD was utilized. This model was composed of rat liver-specific biomatrix scaffolds and human hepatic cells, induced by a high-fat medium. Its biological characteristics included increased insulin resistance, and elevated urea, albumin and CYP450, consistent with the clinical features of human NAFLD [15,25,26]. We found baicalin significantly reduced hepatocyte injury in the FFA group but the mechanism remains unclear. Previous findings suggested that baicalin combined with puerarin and berberine could reduce liver lipid accumulation and improve liver function [27]. In addition, baicalin alone activates hepatic carnitine palmitoyltransferase 1 (CPT1) to ameliorate diet-induced obesity and hepatic steatosis [28]. However, the actual damage to hepatocytes is the second hit in the “two-hit” theory of NAFLD, a consequence of increased ROS production in the liver due to increased metabolism of FFA, and promoting mitochondrial dysfunction, the upregulation of proinflammatory cytokines and hepatic stellate cell activation [29]. In this study, baicalin reduced ROS levels in the FFA group and lipid peroxidation, reflected by MDA. Meanwhile, it also increased the contents of antioxidants, including SOD and GSH. All these changes decreased the cytotoxicity induced by peroxidation to a certain degree.

The effects of baicalin on oxidative stress also conferred mitochondrial protection. It is known that mitochondria play an important role in the occurrence and development of oxidative stress. Increased oxidative stress can damage mitochondria, and subsequent mitochondrial dysfunction increases the accumulation of mitochondrial ROS, leading to cell damage [19]. Mitochondrial damage may be the basis of the development of NAFLD because it changes the energy homeostasis and stimulates the production of ROS [30]. MMP destruction is the main marker of mitochondrial dysfunction. MMP loss leads to the loss of the mitochondrial electron transport chain, reduced metabolic oxygen consumption, ATP depletion and low-energy metabolism [31]. Indeed, in the FFA group, MMP and ATP5A expression were both significantly decreased due to excessive ROS, and the amounts of mitochondria were reduced under an electron microscope. Baicalin treatment significantly reversed MMP and ATP5A expression, indicating an amelioration of mitochondrial function. In addition, oxidative stress on the mitochondria can induce permeabilization and cytochrome c release, triggering the mitochondrial apoptotic pathway [19]. In the present study, the protective effect of baicalin on the mitochondria also inhibited FFA-induced apoptosis, evidenced by decreased TUNEL expression and cleaved caspase-3 expression. Therefore, baicalin could reduce cell injury and ameliorate hepatocyte function.

How baicalin mediates the antioxidant response in the NAFLD model remains an open question. Nrf2 is the master regulator of the primary factors involved in cellular defense through the mediation of antioxidation [23]. In response to oxidative stress, activated Nrf2 translocates into the nucleus and regulates genes driven by antioxidant response elements, including heme oxygenase-1 and NAD(P)H, glutathione S-transferase (GST) and glutathione peroxidase [32]. Baicalin alleviates oxidative stress via the Nrf2 pathway in diabetic nephropathy and alcoholic liver disease [23,33]. In our study, baicalin upregulated Nrf2 under normal conditions because of its feature of activation on the Nrf2 pathway, which was proved in other studies [33]. However, in the FFA group with or without baicalin treatment, Nrf2 was upregulated, which seems confusing. Upregulation of Nrf2 induced by oxidative stress is positively regulated for cell defense; in this instance, upregulation of Nrf2 induced by baicalin was masked, which suggested the mechanism of baicalin-mediated antioxidation was not mainly dependent on the Nrf2 pathway in the 3D model of NAFLD.

An interesting finding was that baicalin itself had strong antioxidative effects due to its molecular chemical structure, which was considered an important mechanism in this study. Baicalin has many -OH groups that are easily oxidizable by ROS, which could reduce the deleterious effects of ROS on other organelles and alleviate the oxidative stress response to a certain extent. Baicalin performed well in both total antioxidant capacity and superoxide anion scavenging activity, in a dose-dependent manner. From this point of view, baicalin might directly scavenge ROS to reduce the associated damage on mitochondria and cells in the fatty liver. Accordingly, the enhancement of SOD and GSH by baicalin was more likely due to reduced consumption rather than increased synthesis. In addition, baicalin alleviated cell swelling, leukocyte infiltration and carnitine palmitoyltransferase 1 activation to ameliorate hepatic steatosis, all of which contributed to baicalin-induced protection of hepatic cells from necrosis [28,34,35].

## 5. Conclusions

Baicalin attenuates oxidative stress to suppress apoptosis and mitochondrial dysfunction via its own antioxidant capacity in a 3D model of NAFLD. Since oxidative stress regulation and ROS elimination are important directions in liver prevention and NAFLD treatment, this study provides new ideas for the clinical treatment of NAFLD, as well as experimental data regarding the pharmacological effects of baicalin.

## Data Availability

All the raw data are available from the corresponding author upon reasonable request.
